# Integrative Analysis of Dysregulated lncRNA-Associated ceRNA Network Reveals Functional lncRNAs in Gastric Cancer

**DOI:** 10.3390/genes9060303

**Published:** 2018-06-18

**Authors:** Haiming Liu, Zhe Zhang, Nan Wu, Hao Guo, Hao Zhang, Daiming Fan, Yongzhan Nie, Yuanning Liu

**Affiliations:** 1 College of Computer Science and Technology, Jilin University, Changchun 130012, Jilin, China; liuhaiming0702@163.com (H.L.); zhangh@jlu.edu.cn (H.Z.); 2 Key Laboratory of Symbolic Computation and Knowledge Engineering, Ministry of Education, Jilin University, Changchun 130012, Jilin, China; 3 State Key Laboratory of Cancer Biology, National Clinical Research Center for Digestive Diseases, Xijing Hospital of Digestive Diseases, Fourth Military Medical University, Xi’an 710032, Shaanxi, China; zz141421@126.com (Z.Z.); 13119110590@136.com (N.W.); h.guo@foxmail.com (H.G.); fandaim@fmmu.edu.cn (D.F.); 4 College of Life Sciences, Northwest University, Xi’an 710032, Shaanxi, China

**Keywords:** competing endogenous RNA, long non-coding RNA, gastric cancer, network analysis, *DLEU2*, *DDX11-AS1*

## Abstract

Mounting evidence suggests that long noncoding RNAs (lncRNAs) play important roles in the regulation of gene expression by acting as competing endogenous RNA (ceRNA). However, the regulatory mechanisms of lncRNA as ceRNA in gastric cancer (GC) are not fully understood. Here, we first constructed a dysregulated lncRNA-associated ceRNA network by integrating analysis of gene expression profiles of lncRNAs, microRNAs (miRNAs), and messenger RNAs (mRNAs). Then, we determined three lncRNAs (*RP5-1120P11*, *DLEU2*, and *DDX11-AS1*) as hub lncRNAs, in which associated ceRNA subnetworks were involved in cell cycle-related processes and cancer-related pathways. Furthermore, we confirmed that the two lncRNAs (*DLEU2* and *DDX11-AS1*) were significantly upregulated in GC tissues, promote GC cell proliferation, and negatively regulate miRNA expression, respectively. The hub lncRNAs (*DLEU2* and *DDX11-AS1*) could have oncogenic functions, and act as potential ceRNAs to sponge miRNA. Our findings not only provide novel insights on ceRNA regulation in GC, but can also provide opportunities for the functional characterization of lncRNAs in future studies.

## 1. Introduction

Gastric cancer (GC) is one of the leading causes of cancer-related death worldwide, and imposes a considerable global health burden [1,2]. In 2012, an estimated 723,100 deaths from GC and nearly half of the burden occurred in Eastern Asia, particularly in China, Korea, and Japan [3]. In China, GC was the second most common cancer, as reported by the National Central Cancer Registry (NCCR) [4].

Previous studies have demonstrated that coding RNAs and non-coding RNAs (ncRNAs) are closely related to cancer [5]. MicroRNAs (miRNAs) are an important subclass of endogenous non-coding RNAs with a typical length of 23 nucleotides [6], and play crucial roles in cancer by regulating gene expression at the post-transcriptional level [7]. Long non-coding RNAs (lncRNAs), another important subclass of endogenous non-coding RNAs, are longer than 200 nucleotides, and lack protein-coding capability [8]. Similar to coding RNAs and miRNAs, some lncRNAs can drive cancer phenotypes [9]. However, the complete spectrum of mechanisms by which lncRNAs affect cancer development and pathology remains to be fully elucidated.

Recent studies have demonstrated that lncRNA can act as a miRNA sponge via competing endogenous RNA (ceRNA) activity [10,11]. Understanding this novel RNA crosstalk will lead to significant insights into gene regulatory networks and have implications in human development and disease [12]. For example, *PTENP1*, a pseudogene of the tumor-suppressor gene *PTEN*, is among the first reported miRNA sponges with a function in cancer [13]. *BRAF* is an oncogene and its overexpression leads to enhanced activity of the MAPK signaling pathway [14]. Overexpression of the *BRAF* pseudogene results in upregulation of *BRAF*. *HOTAIR* acts as an miRNA sponge to regulate *HER2* expression by sponging hsa-miR-331-3p in GC [15]. *GAPLINC* acts as a ceRNA for hsa-miR-211-3p, and mediates cell migration and proliferation in GC [16]. *KRTAP5-AS1* and *TUBB2A* participate in the regulatory network of *CLDN4* based on ceRNA activity in GC [17]. *MEG3* can acts as a ceRNA to regulate GC progression [18].

Based on the ceRNA hypothesis, comprehensive analyses of lncRNA-associated ceRNA networks have been reported in ovarian cancer [19], glioblastoma [20], prostate cancer [10], thyroid carcinoma [21], and breast cancer [22]. Xia et al. [23] constructed a cancer-associated ceRNA network in GC based on lncRNA microarray data. Li et al. [24] showed the GC-specific lncRNA expression patterns, and constructed a ceRNA network based on The Cancer Genome Atlas (TCGA). Although previous studies have identified ceRNAs in GC, the functional roles and regulatory mechanisms of lncRNAs as ceRNAs in GC are not fully understood.

In this study, we first systematically integrated gene expression profiles of GC, miRNA target interactions, and significantly co-expressed gene pairs based on the ceRNA hypothesis. We then constructed dysregulated lncRNA-associated ceRNA network. After the network topological analysis, we determined that the hub lncRNAs play crucial roles in GC development. By reconstructing and functionally analyzing the hub lncRNA-associated subnetworks, we identified three hub lncRNAs (*RP5-1120P11*, *DLEU2*, and *DDX11-AS1*) that are involved in cell cycle-related processes and cancer-related pathways. Finally, we validated that *DLEU2* and *DDX11-AS1* are significantly upregulated in GC tissues by using qRT-PCR. Cellular experimental results indicated that the two lncRNAs promote GC cell proliferation and negatively regulate miRNA expression. Through an integrative analysis of dysregulated lncRNA-associated ceRNA network (Figure S1), we determined that hub lncRNAs (*DLEU2* and *DDX11-AS1*) have oncogenic functions, and act as potential ceRNAs to sponge miRNA. Our findings can be used to reveal ceRNA regulation in GC, and provide novel insights on functional characterization of lncRNAs.

## 2. Materials and Methods

### 2.1. GDC Data Collection and Processing

Gastric cancer gene expression data were obtained from Genomic Data Commons (GDC, https://cancergenome.nih.gov/) [25]. The upper quantile normalized FPKM (fragments per kilobase per million) values and the normalized count in reads-per-million-miRNA-mapped values were used. Patients with follow-up time exceeding three years were excluded. Overall, a total of 364 samples, including 337 GC samples and 27 normal samples, were included in our study and each sample consisted of the corresponding RNA-seq data and miRNAseq data.

To assemble a lncRNA-associated ceRNA data, we downloaded the RNA gene annotation and sequence data from GENCODE v26 (https://www.gencodegenes.org/), which aims to identify all gene features in the human genome using a combination of computational analysis, manual annotation, and experimental validation [26]. Transcripts longer than 200 nt and categorized as “lincRNA”, “antisense”, “processed_transcript”, and “non_coding” were identified as lncRNAs. Genes categorized as “protein_coding” were considered messenger RNAs (mRNAs). In total, 19,799 mRNAs and 13,545 lncRNAs in 364 samples were annotated

Genes expressed in more than 90% of samples were retained, and the values of zero or NA in the expression profile were replaced with the (minimum non-zero FPKM value)/2 of the corresponding genes. All the expression profile values were then log2 transformed. Therefore, the expression profiles of 16,363 mRNAs, 440 miRNAs, and 4211 lncRNAs in 364 samples were selected for further analysis.

### 2.2. Identifying Differentially Expressed mRNAs, lncRNAs, and miRNAs Related to GC

To identify differentially expressed lncRNAs (DELs), mRNAs (DEMs), and miRNAs (DEMis), we used the two-tailed Mann–Whitney U test to compare the expression values in cancer with those in normal. For all *p*-values, FDR (false discovery rate) was used to correct the statistical significance of multiple testing by the Benjamini–Hochberg method. In this paper, we mainly focused on the upregulated lncRNAs, upregulated mRNAs, and downregulated miRNAs, which could have oncogenic functions based on ceRNA activity. Therefore, we defined differentially expressed lncRNAs/mRNAs with fold change ≥1 and adjusted *p*-value <0.001 as DELs/DEMs. In order to avoid insufficient number of downregulated miRNAs, we defined differentially expressed miRNAs with fold change ≤−0.6 and adjusted *p*-value <0.001 as DEMis.

### 2.3. Identification of miRNA–Target Interactions

MicroRNA sequences were obtained from the TargetScan website (http://www.targetscan.org/) [27]. LncRNA sequences were obtained from the GENCODE v26. miRNA–mRNA interactions were obtained from three reliable online miRNA-target databases: miRWalk 2.0 [28], miRTarBase 6.0 [29], and DIANA-TarBase v7.0 [30]. miRNA–lncRNA interactions were predicted using TargetScan 7.0 [27] with default parameters. After integrating the above methods, a total of 148,459 non-redundant reliable miRNA–mRNA interactions and 29,523 miRNA–lncRNA predicted interactions were obtained. We then selected 1146 reliable DEM–DEMi interactions and 2794 predicted DEL–DEMi interactions for further analysis.

### 2.4. Identification of Potential ceRNA Triples Based on Gene Expression

The competition of lncRNAs with mRNAs to sponge miRNAs is reflected at the gene expression level [31]. These lncRNA–miRNA–mRNA interactions were defined as ceRNA triples, and the potential ceRNA triples were identified as follows: (1) expression correlations among all mRNAs, lncRNAs, and miRNAs in GC were evaluated by the Pearson correlation coefficient (PCC). The PCC of gene pair (X and Y) is defined as

(1) $$PCC\left(X,Y\right)=\frac{1}{n-1}\sum_{i=1}^{n}\left(\frac{Exp\left(X,i\right)-\bar{Exp}\left(X\right)}{\sigma \left(X\right)}\right)\cdot \left(\frac{Exp\left(Y,i\right)-\bar{Exp}\left(Y\right)}{\sigma \left(Y\right)}\right),$$

where n is the number of GC cancer samples; Exp(X,i) (Exp(Y,i)) is the expression level of X (Y) in sample i; $\bar{Exp}\left(X\right)$ (Exp(Y,i)) denotes the average expression level of X (Y), and σ(X) (σ(Y)) represents the standard deviation of expression level of X (Y). In order to obtain the distribution of the overall PCC in GC, we calculated the PCC between 16,363 mRNAs, 440 miRNAs, and 4211 lncRNAs, respectively. We then obtained the PCC of 68,904,593 mRNA–lncRNA pairs, 1,852,840 lncRNA–miRNA pairs, and 7,199,720 mRNA–miRNA pairs. (2) To obtain the significant co-expressed pairs, PCC higher than the threshold of 95% was considered a significant positively co-expressed pair, and lower than the threshold of 95% was considered as a negatively co-expressed pair. Therefore, DEL–DEM pairs with PCC > 0.292 and *p*-value < 0.0001 was considered as significantly co-expressed DEL–DEM pairs (Figure S2A). DEM–DEMi pairs with PCC <−0.275 and *p*-value <0.0001 were considered as significantly negatively co-expressed DEM–DEMi pairs (Figure S2B). DEL–DEMi pairs with PCC < −0.233 and *p*-value < 0.0001 were considered as significantly negatively DEL–DEMi pairs (Figure S2C). (3) For a given significant co-expressed DEM–DEL pair, both DEM and DEL interacted with the same DEMi, and were negatively co-expressed with the same DEMi, and this DEL–DEMi–DEM interaction was defined as a potential ceRNA triple.

### 2.5. Construction Network and Topological Analysis

To clarify the roles of the dysregulated lncRNA-associated ceRNA network, we constructed a co-expression network of DEL–DEMi–DEM interactions based on the potential ceRNA triples, and visualized this ceRNA network by Cytoscape 3.5.1 [32]. Betweenness centrality (BC) is a measure of centrality in a network based on the number of shortest paths from every node to all others passing through this node. In a network, a node with a higher BC is implied to act as a bridge to connect different nodes and control the network communications. In this study, BC was considered as the topological analysis measure to select the hub lncRNAs. The BC of a node i is defined as

(2) $$Betweenness\;centrality=\sum_{s\neq i\neq t}\frac{p_{st}\left(i\right)}{p_{st}},$$

where $p_{st}$ is the total number of the shortest paths from node s to node  t, and $p_{st}\left(i\right)$ is the number of these paths that pass through node i. 

We assumed that the hub lncRNAs play critical roles in the ceRNA network. Accordingly, we reconstructed the hub lncRNA-associated subnetwork with the nodes, which is the hub lncRNA related to the first-neighbor miRNAs and the second-neighbor mRNAs in the ceRNA network.

### 2.6. Functional Enrichment Analysis

To reveal the function of lncRNA-associated ceRNA network, the DEMs were subjected to functional enrichment analysis via gene ontology (GO) and Kyoto Encyclopedia of Genes and Genomes (KEGG) with the R package cluster Profiler [33]. GO is a structured standard biological model established by the GO consortium, which aims to establish a standard system for computing the functions of genes, covering biological processes (BP), molecular functions (MF), and cellular components (CC) [34]. KEGG is widely used as a reference for integrating large-scale molecular datasets generated by sequencing and high-throughput experimental technologies [35]. In this study, we considered functional categories with a *p*-value < 0.05.

### 2.7. Patient Samples and GC Cell Lines

Gastric cancer tissues and adjacent normal tissues were collected from patients who underwent surgery treatment at the Xijing Hospital (Xi’an, China). Written informed consents had been obtained from all patients who donated tissue samples, which was under the supervision of the Hospital’s Protection of Human Subjects Committee. None of our study patients had received chemotherapy or radiotherapy before the surgery, and all tissues were paraffin-embedded after RNA and protein isolation. Human GC cell lines BGC823 and MKN45 were purchased from the Cell Bank of the Chinese Academy of Sciences. Cells were incubated at 37 °C in a humidified atmosphere containing 5% CO_2_ and cultured in Dulbecco’s Modified Eagle’s Medium (ThermoFisher Scientific, Waltham, MA, USA) combined with 10% fetal bovine serum (FBS).

### 2.8. RNA Isolation and Real-Time PCR Analysis

Total RNA was isolated using a Qiagen miRNeasy kit (Qiagen, Hilden, Germany) according to the manufacturer’s protocol. Then, reverse transcription of RNA to cDNA was performed using the the Prime Script RT Master Mix (TaKaRa Bio Inc., Kusatsu, Japan). qRT-PCR was carried out using SYBR premix Ex Taq (Takara Bio Inc., Kusatsu, Japan). RNA qRT-PCR PrimerSets specifc for *DLEU2*, *DDX11-AS1*, hsa-miR-30-5p, and hsa-miR-145-5p were designed by RiboBio (Guangzhou, China). The expression levels of the RNA and internal control (U6) were measured using qRT-PCR (Bio-Rad, Hercules, CA, USA).

### 2.9. RNA Interference by siRNA

The siRNA targeting lncRNAs and siRNA control were synthesized by RiboBio (Guangzhou, China). The sequences of siRNAs were as follows: *DLEU2*-si1: 5′-CTAATAGCTTGAACCCTTT-3′; *DLEU2*-si2: 5′-CTAACTCCTCCACCAGAAA-3′; *DDX11-AS1*-si1: 5′-CTCATTCCTCTGCCTACAA-3′; *DDX11-AS1*-si2: 5′-AGACTCATGAGCTGAAGAT-3′. BGC823 and MKN45 cells cultured in 6-well plates were transfected with the siRNA according to the manufacturer’s instructions. Cells were harvested at 36 h after transfection. The specific silencing of the two lncRNAs expression was assessed by using qRT-PCR.

### 2.10. CCK-8 and Colony Formation Assays

Cells were seeded in 96-well plates (1 × 10^4^ cells per well), cultivated at 37 °C in a 5% CO_2_ humidified incubator for 24 h. Ten microliters of Cell Counting Kit-8 solution (CCK-8) (Dojindo Laboratories, Kumamoto, Japan) were added to each well and incubated at 37 °C in a 5% CO_2_ humidified incubator for 2 h. Spectrometer Varioskan Flash (ThermoFisher Scientific) was used to measure the 450 nm absorbance. The CCK-8 assay was performed every 24 h for 5 days. A proliferation curve was drawn with the time as the abscissa, and average absorbance value in each group as the ordinate. For the colony formation assay, Cells were seeded in 6-well plates (1 × 10^3^ cells per well), and were incubated at 37 °C in a 5% CO_2_-humidified incubator. After 2 weeks, the cells were stained with Gentian violet (Beyotime Biotechnology, Shanghai, China). Triplicate reactions were performed for the experiment.

### 2.11. Statistical Analysis of Biological Experimental Results

Biological experimental results were analyzed using paired or unpaired *t*-test (GraphPad Software, La Jolla, CA, USA). Values of *p* < 0.05 were considered statistically significant (*). ALL biological experimental results were presented as mean ± SD.

## 3. Results

### 3.1. Identification of Differentially Expressed lncRNAs, mRNAs, and miRNAs

**Figure 1:**
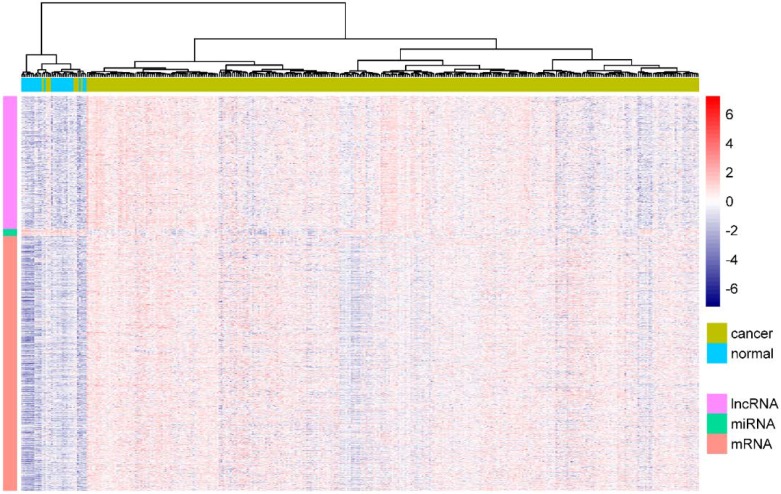
The heatmap of lncRNAs (DELs), mRNAs (DEMs), and miRNAs (DEMis) in gastric cancer (GC). The samples were represented in columns, and the genes were represented in rows, with different colors. The expression value for each row was normalized by the z-score. Red indicates high relative expression and blue indicates low expression of genes as shown in the scale bar.

We obtained the gene expression profiles of 337 GC samples and 27 normal samples from GDC and identified differentially expressed genes. A total of 757 lncRNAs, 2441 mRNAs, and 256 miRNAs were detected. Among them, 571 lncRNAs, 1097 mRNAs, and 227 miRNAs were significantly upregulated, while 186 lncRNAs, 1344 mRNAs, and 29 miRNAs were significantly downregulated in GC (Table S1). In this paper, we mainly focused on the upregulated lncRNAs, upregulated mRNAs, and downregulated miRNAs, which could have oncogenic functions based on ceRNA activity. Therefore, we selected 571 DELs, 1097 DEMs, and 29 DEMis from the significantly differentially expressed set for further analysis. To determine if the DELs, DEMs, and DEMis can be used to separate normal samples from cancer samples, we utilized “ward.D” algorithm and Euclidean distance to cluster these genes with the R package pheatmap. The heatmap indicated that all samples could be clearly separated into two groups, and less than 3% of the cancer samples were incorrectly classified (Figure 1). These differentially expressed genes were therefore considered as potential key regulators to construct the ceRNA network in GC.

### 3.2. Construction and Functional Analysis of ceRNA Network in GC

**Figure 2:**
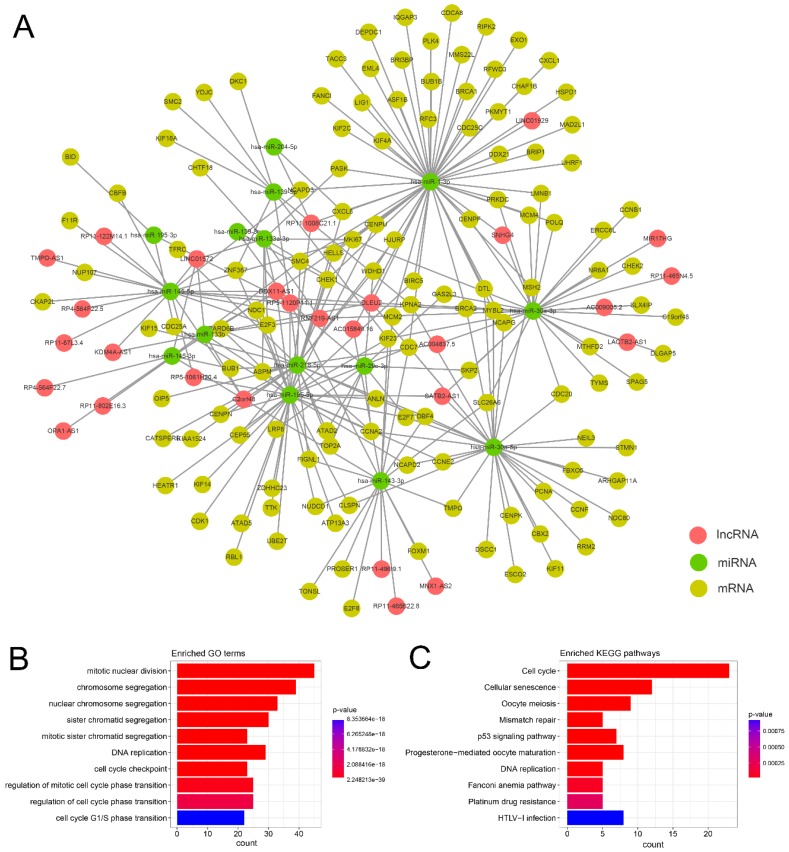
Global view and function analysis of the ceRNA network in GC. (**A**) Global view of the ceRNA network in GC. The lncRNA, mRNA, and miRNA were colored red, yellow, and green. There were 28 lncRNAs, 130 mRNAs, 16 miRNAs, and 173 edges in this network; (**B**) The top 10 significant gene ontology (GO) terms in GC-related ceRNA network; (**C**) The top 10 significant Kyoto Encyclopedia of Genes and Genomes (KEGG) pathways in GC-related ceRNA network. The count indicates the number of genes associated with a GO term in biological processes (BP) or a KEGG pathway.

To construct the ceRNA network in GC, we focused on the relationships among the DELs, DEMs and DEMis. First, we identified miRNA–target interactions by integrating reliable online databases and prediction software. We then selected 1146 reliable DEM–DEMi interactions from 148,459 miRNA–mRNA interactions and 2794 candidate DEL–DEMi interactions from 29,523 miRNA–lncRNA interactions for further analysis. To reduce false positives, we used the PCC to filter the significant co-expressed DEL–DEMi–DEM interactions. After filtering the interactions, a total of 442 potential ceRNA triples were identified (Table S2). Based on the potential ceRNA triples, we constructed and visualized a dysregulated lncRNA-associated ceRNA network by Cytoscape (Figure 2A). The ceRNA network was a fully connected network, which consisted of 174 nodes (including 28 DELs, 130 DEMs, and 16 DEMis) and 173 edges. Then, we utilized the DEMs to reveal the gene functions of the dysregulated lncRNA-associated ceRNA network via the functional enrichment analysis. The ceRNA function analysis revealed 250 GO terms in BP and 12 KEGG pathways (Figure 2B,C, and Table S3). Cancer is characterized by aberrant cell cycle activity [36], and most of these GO terms are cell cycle process related in the ceRNA network. Among the KEGG pathways, cell cycle, p53 signaling pathway, Fanconi anemia pathway, mismatch repair, DNA replication, and platinum drug resistance are cancer-related pathways [36,37,38,39,40].

### 3.3. Topological Analysis of the lncRNA-Associated ceRNA Network in GC

**Figure 3:**
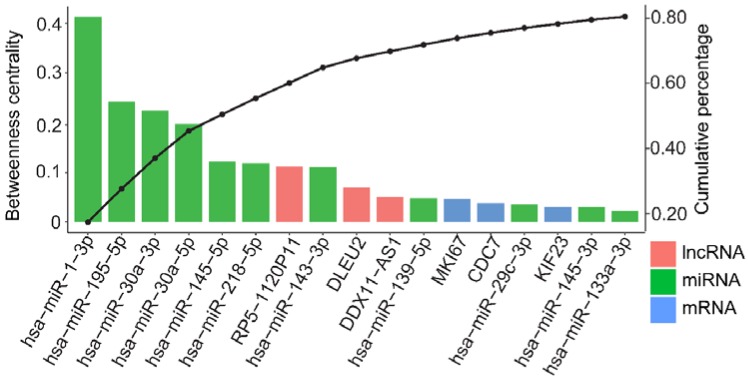
The top highest 17 BC nodes in ceRNA network. The left Y-axis denotes the betweenness centrality (BC) value of node, and the right Y-axis denotes the cumulative percentage of the BC. These nodes made up 80.5% of all the BCs in the ceRNA network.

To clarify the roles of the dysregulated lncRNA-associated ceRNA network, we computed BC, which are topological features of ceRNA network, as shown in Table S4. A node with a higher BC plays a more important role in the ceRNA network, and is more likely to be associated with cancer [19,41]. Among the 174 nodes in the ceRNA network, the top 17 highest BC nodes (including 3 DELs, 3 DEMs, and 11 DEMis) were shown in Figure 3. We selected the three DELs (*RP5-1120P11*, *DLEU2*, and *DDX11-AS1*) with the highest BC values as the hub lncRNAs, and calculated the hub lncRNAs related to the first-neighbor DEMis, the second-neighbor DEMs, and the ceRNA triples. We determined that the hub lncRNAs were related to 87.5% of the DEMis, 87.69% of the DEMs, and 53.85% of the ceRNA triples in the ceRNA network (Table 1). These results indicated that the hub lncRNAs are potential key regulators controlling the ceRNA network in GC.

### 3.4. Reconstruction and Functional Analysis of Hub lncRNA-Associated Subnetworks in GC

**Figure 4:**
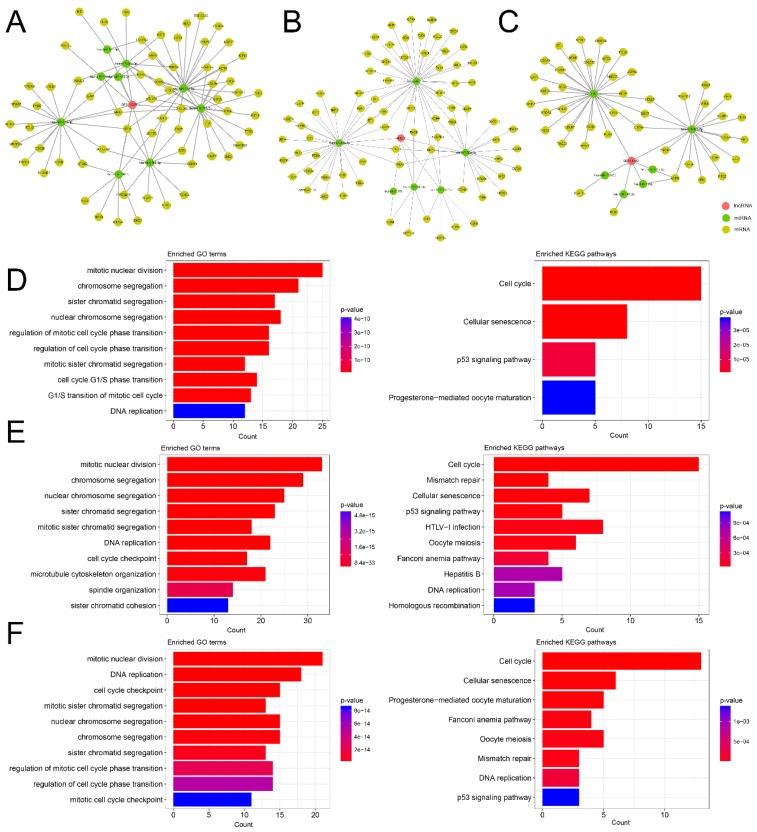
The ceRNA subnetworks and functional enrichment analysis of the hub lncRNAs. (**A**) The *RP5-1120P11*-associated ceRNA subnetwork; (**B**) *DLEU2*-associated ceRNA subnetwork; (**C**) *DDX11-AS1*-associated ceRNA subnetwork. The lncRNA, mRNA, and miRNA were colored red, yellow, and green; (**D**) The enrichment analysis result of *RP5-1120P11*-associated ceRNA subnetwork; (**E**) The enrichment analysis result of *DLEU2*-associated ceRNA subnetwork; (**F**) The enrichment analysis result of *DDX11-AS1*-associated ceRNA subnetwork. The count indicates the number of genes associated with a GO term in BP or a KEGG pathway.

We assumed that the hub lncRNAs (*RP5-1120P11*, *DLEU2*, and *DDX11-AS1*) play critical roles in the GC-related ceRNA network. Accordingly, we reconstructed the hub lncRNA-associated subnetworks with the nodes, which were the hub lncRNAs related to the first-neighbor miRNAs and the second-neighbor mRNAs in the ceRNA network. To reveal the functions of the hub lncRNAs, we performed functional enrichment analysis for each associated subnetwork (Figure 4).

*RP5-1120P11* is the most important key regulator in the ceRNA network. *RP5-1120P11* interacts with 9 DEMis (hsa-miR-139-3p, hsa-miR-139-5p, hsa-miR-143-3p, hsa-miR-145-5p, hsa-miR-195-3p, hsa-miR-195-5p, hsa-miR-218-5p, hsa-miR-29c-3p, and hsa-miR-30a-3p) and is co-regulated with 64 DEMs (Figure 4A). The function analysis of *RP5-1120P11*-associated subnetwork revealed 160 GO terms in BP and 4 KEGG pathways (Figure 4D, Table S3). We determined that most of these GO terms and KEGG pathways are cell cycle process-related and cancer-related. However, there is no experimental evidence to support the function of *RP5-1120P11* in recent studies.

**Figure 5:**
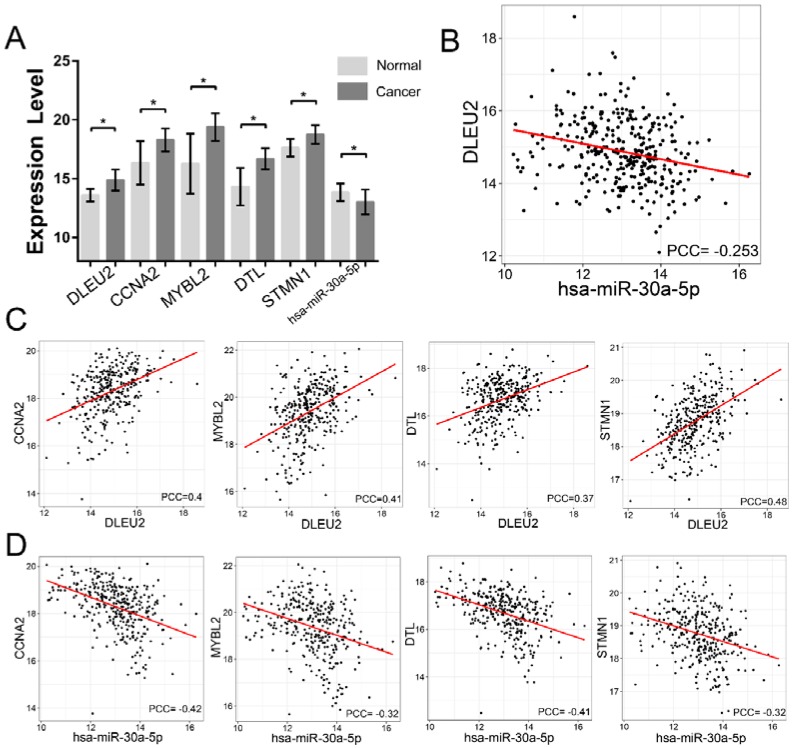
The expression levels and correlation of *DLEU2* related DEMi and DEMs in GC. (**A**) The boxplot showed the expression levels of *DLEU2* and *DLEU2* related DEMs. * denotes *p* < 0.001; (**B**) The scatter plots showed the correlation of expression between *DLEU2* and hsa-miR-30-5p; (**C**) The scatter plots showed the correlation of expression between *DLEU2* and *DLEU2* related DEMs; (**D**) The scatter plots showed the correlation of expression between *DLEU2* related DEMs and hsa-miR-30-5p. All the data were obtained from GDC.

*DLEU2* is a lymphocytic leukemia-related gene and the host gene promoter for miR-15a/16-1 [42]. We observed that *DLEU2* was co-regulated with 77 DEMs by acting as a ceRNA to sponge 6 DEMis (hsa-miR-1-3p, hsa-miR-30a-3p, hsa-miR-30a-5p, hsa-miR-133a-3p, hsa-miR-133b, and hsa-miR-145-5p) (Figure 4B). The functional analysis of *DLEU2*-associated subnetwork revealed 238 GO terms in BP and 11 KEGG pathways (Figure 4E, Table S3). We determined that most of these GO terms are cell cycle process-related, and several pathways are cancer-related pathways, such as cell cycle, mismatch repair, p53 signaling pathway, Fanconi anemia pathway, and DNA replication. Intriguingly, *DLEU2* downregulates hsa-miR-30-5p by ceRNA activity in previous study [43]. In the *DLEU2*-associated ceRNA network, hsa-miR-30a-5p is downregulated in GC, and negatively co-expressed with *DLEU2* (Figure 5A,B). In addition, many studies have reported that hsa-miR-30a-5p is a tumor suppressor in GC [44,45], and recent studies have shown that *CCNA2*, *MYBL2*, *DTL*, and *STMN1* are regulated by hsa-miR-30a-5p [46,47,48,49]. In the network, we determined that *CCNA2*, *MYBL2*, *DTL*, and *STMN1*, which are significantly upregulated in GC (Figure 5A), are co-expressed with *DLEU2* (Figure 5C) and negatively co-expressed with the same hsa-miR-30a-5p (Figure 5D). Therefore, we suspected that some ceRNA triples, such as *DLEU2*–hsa-miR-30a-5p–*CCNA2*, *DLEU2*–hsa-miR-30a-5p–*MYBL2*, *DLEU2*–hsa-miR-30a-5p–*DTL*, and *DLEU2*–hsa-miR-30a-5p–*STMN1*, are potential therapeutic targets in GC.

*DDX11-AS1* is upregulated in multiple cancer types and affects sister chromatid cohesion [50]. The subnetwork related to *DDX11-AS1* consisted of 6 DEMis and 45 DEMs (Figure 4C). The function analysis of the *DDX11-AS1*-associated subnetwork revealed 204 GO terms in BP and 8 KEGG pathways (Figure 4F, Table S3). We determined that most of these GO terms are cell cycle process-related and most pathways are cancer-related pathways, such as cell cycle, Fanconi anemia pathway, mismatch repair, DNA replication, and p53 signaling pathway.

All these results will provide us important cell cycle processes and cancer-related pathways information for understanding lncRNA-associated ceRNA network in GC.

### 3.5. DLEU2 and DDX11-AS1 Are Upregulated in GC Tissues, Promote GC Cell Proliferation, and Inhibit miRNA Expression, Respectively

**Figure 6:**
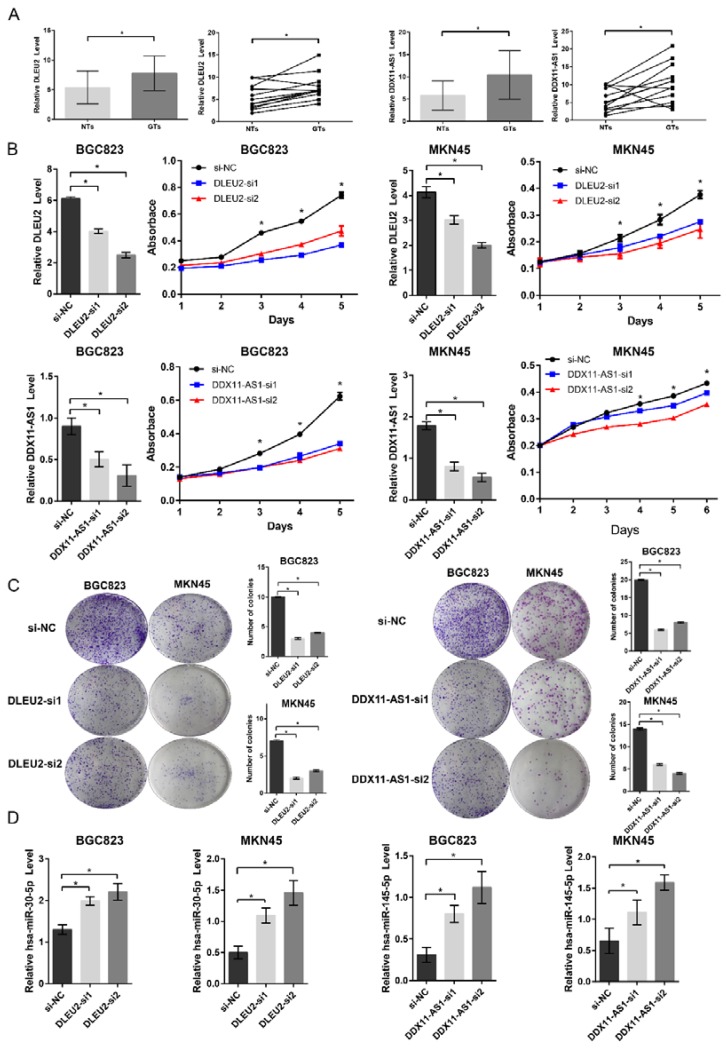
Functional validation of the predicted hub lncRNAs in GC. (**A**) The qRT-PCR results showed that the expression levels of *DLEU2* and *DDX11-AS1* in 12 pairs of GC tissues (GTs) were significantly higher than the adjacent normal tissues (NTs); (**B**) The qRT-PCR results showed *DLEU2* and *DDX11-AS1* knockdown in BGC823/MKN45 cells after transfection with NC (negative control), siRNA 1 (si-1), and siRNA 2 (si-2). The CCK-8 assays showed that knockdown of *DLEU2* and *DDX11-AS1* suppressed cell proliferation in BGC823/MKN45 cells; (**C**) The colony-forming assays showed that knockdown of *DLEU2* and *DDX11-AS1* suppressed cell proliferation in BGC823/MKN45 cells; (**D**) In BGC823 and MKN45 cells, knockdown of *DLEU2* and *DDX11-AS1* increased hsa-miR-30-5p and hsa-miR-145-5p expression, respectively. Biological experimental results were analyzed using paired or unpaired *t*-test, * denotes *p* < 0.05.

To validate the findings, we first examined *DLEU2* and *DDX11-AS1* expression level in a cohort of 12 paired GC tissues, and compared with those of the adjacent normal tissues by using qRT-PCR. The *DLEU2* and *DDX11-AS1* expression levels were significantly upregulated in GC tissues (Figure 6A). siRNA-mediated silencing of lncRNAs were validated by qRT-PCR, and the knockdown efficiencies were achieved in BGC823 and MKN45 cell lines (Figure 6B). Next, we investigated the potential effect of *DLEU2* and *DDX11-AS1* in both cell lines. Knockdown of *DLEU2* and *DDX11-AS1* suppressed cell proliferation in BGC823 and MKN45 cells, as determined by CCK-8 assays (Figure 6B). The results of colony formation assay showed that BGC823 and MKN45 cells with *DLEU2* and *DDX11-AS1* knockdown formed significantly less colonies than control cells (Figure 6C). By miRNA target prediction, hsa-miR-30a-5p and hsa-miR-145-5p were noticed, as they have one putative target on *DLEU2* and *DDX11-AS1*. *DLEU2* and *DDX11-AS1* complementarily matched the sequences on miRNA seed position, respectively. hsa-miR-30-5p regulated *DLEU2* [43] and is a tumor suppressor in GC [44,45]. hsa-miR-145-5p inhibits gastric cancer cell migration and metastasis [51], and the relationship between *DDX11-AS1* and hsa-miR-145-5p has not been reported in previous studies, but the correlation coefficient between *DDX11-AS1* and hsa-miR-145-5p was the most significant (Table S2). Therefore, we selected hsa-miR-30-5p and hsa-miR-145-5p to verify the relationships between lncRNAs and miRNAs. By using qRT-PCR, we observed that knockdown of *DLEU2* and *DDX11-AS1* significantly increased the hsa-miR-30-5p and hsa-miR-145-5p expression level, respectively (Figure 6D). These results indicated that *DLEU2* and *DDX11-AS1* might have oncogenic function and act as potential ceRNAs to sponge miRNAs in GC.

## 4. Discussion

Gastric Cancer is a leading cause of cancer-related death worldwide, and the second most common cancer in China. Many studies have demonstrated that coding RNAs and ncRNAs are closely related to GC, but the mechanism of GC remains to be elucidated. According to the ceRNA hypothesis, lncRNAs can act as miRNA sponges to construct a complex ceRNA network with miRNAs and mRNAs, as reported in many cancer types [10,19,20,22,23,31]. Thus ceRNAs play an important role in multilayered gene regulation and contribute to cancer development and pathology [52]. Some studies have constructed lncRNA-associated ceRNA networks in GC [23,24], but the mechanism of lncRNAs as ceRNAs in GC remains poorly explored.

In this study, we systematically integrated gene expression profiles and identified 571 DELs, 1097 DEMs, and 29 DEMis in GC. We determined that top DELs, such as *FEZF1-AS1* [53], *HOTAIR* [54], *HOXA11-AS* [55], and *HOTTIP* [56], were GC-related lncRNAs, top DEMs such as *CST1* [57], *HOXC10* [58], *HOXA13* [59], and *HOXA11* [60] were GC-related coding RNAs, top DEMis such as hsa-miR-133b, hsa-miR-133a-3p [61], hsa-miR-1-3p [62], and hsa-miR-204-5p [63] were GC-related miRNAs. These differentially expressed genes were considered as potential key regulators in GC.

In this paper, we mainly focused on upregulated lncRNAs in GC samples based on ceRNA activity. Since the expression of lncRNAs are generally lower expressed than protein-coding genes [64], it is difficult to confirm the functions of the low expressed lncRNA in normal samples by experimental methods. On the other hand, the number of GC samples is much larger than controls. It is very difficult to compare a GC-related ceRNA network to a control ceRNA network. To construct the dysregulated ceRNA network in GC, we first identified miRNA–target interactions by integrating reliable online databases and prediction software. To reduce false positives, we used the PCC to filter the significantly co-expressed DEL–DEMi–DEM interactions. We then constructed a ceRNA network based on 442 potential ceRNA triples. The gene expression data and bioinformatics methods used differ from those employed in previous studies, the ceRNA triples were no overlap with others. After network topological analysis, we determined that the hub lncRNAs play crucial roles in GC. By reconstructing and functionally analyzing each hub lncRNA-associated subnetworks, we identified that three hub lncRNAs (*RP5-1120P11*, *DLEU2*, and *DDX11-AS1*) are involved in cell cycle related processes and cancer-related pathways. Although only a few studies of the three lncRNAs were available, some indirect evidence might support their potential regulatory function in GC. *RP5-1120P11* might regulate many cancer-related signaling pathways and is associated with the poor prognosis in ovarian cancer [65]. *DLEU2* is a lymphocytic leukemia-related gene and is the host gene promoter for miR-15a/16-1 [42], and acts as ceRNA to sponge miRNA in clear cell renal cell carcinoma (ccRCC) [43]. *DDX11-AS1*, which is upregulated in multiple cancer types, affects sister chromatid cohesion [50], and could play an important role in hepatocellular carcinoma (HCC) [66]. However, in previous studies, none of them has been reported as functional lncRNAs or act as ceRNAs in GC.

To validate our findings, experimental results revealed that *DLEU2* and *DDX11-AS1* might have oncogenic functions and act as potential ceRNAs to sponge miRNAs in GC. In the lncRNA-associated ceRNA network, hsa-miR-30a-5p is downregulated in GC, and negatively co-expressed with *DLEU2* (Figure 5A,B). Cellular experimental results indicated that hsa-miR-30a-5p is inhibited by *DLEU2* (Figure 6D). Intriguingly, *DLEU2* downregulates hsa-miR-30-5p by ceRNA activity in ccRCC [43]. In addition, many studies have reported that hsa-miR-30a-5p is a tumor suppressor in GC [44,45], and recent studies have shown that *CCNA2*, *MYBL2*, *DTL*, and *STMN1* are regulated by hsa-miR-30a-5p. *CCNA2*, which mediates cell proliferation and tumorigenesis [67], is regulated by miR-29a, miR-30a, and miR-141 to reactivate postnatal cardiomyocyte proliferation [46]. *MYBL2*, which is involved in tumorigenesis, has been implicated in cell proliferation and amplification. *MYBL2* overexpression is associated with lower expression of miR-30a [47]. Overexpression of *DTL*, which plays a crucial role in tumor cell proliferation, is associated with poor survival rates and in GC [68]. *DTL* is regulated by hsa-miR-30a-5p to affect tumor growth in colon carcinoma [48]. *STMN1*, which is associated with poor prognosis in GC patients [69], is regulated by hsa-miR-30a in ovarian papillary serous carcinoma and ovarian clear cell carcinoma [49]. Therefore, we suspected that some ceRNA triples, such as *DLEU2*-hsa-miR-30a-5p-*CCNA2*, *DLEU2*-hsa-miR-30a-5p-*MYBL2*, *DLEU2*-hsa-miR-30a-5p-*DTL*, and *DLEU2*-hsa-miR-30a-5p-*STMN1*, are potential therapeutic targets in GC. Furthermore, hsa-miR-145-5p inhibit gastric cancer cell migration and metastasis [51], and we observed that knockdown of *DDX11-AS1* significantly increased the hsa-miR-145-5p expression level, which means that *DDX11-AS1* could act as potential ceRNA to sponge miRNA. However, the evidence of lncRNA inhibit miRNA expression, which we obtained by qRT-PCR, was indirect. Additional biological experiments will be required to further validate our findings.

These findings indicate that the hub lncRNA plays complex and critical roles in the development and pathology of GC. Although our study provides multistep computational and experimental methods to reveal functional lncRNAs in GC, biological experimental methods are still needed.

## 5. Conclusions

In summary, we employed multistep computational and experimental methods to reveal functional lncRNAs in GC based on the ceRNA hypothesis. By constructing and functionally analyzing lncRNA-associated network, we identified that three hub lncRNAs (*RP5-1120P11*, *DLEU2*, and *DDX11-AS1*) that are involved in cell cycle-related processes and cancer-related pathways. Furthermore, we confirmed that *DLEU2* and *DDX11-AS1* have oncogenic function and act as potential ceRNAs to sponge miRNAs. Our findings not only provide novel insights on ceRNA regulation in GC, but can also provide opportunities for the functional characterization of lncRNAs in future studies.

## Figures and Tables

**Table 1:** The number of hub lncRNA network-related DEMis, DEMs, and ceRNA triples.

Associated Network	No. of DEMis	No. of DEMs	No. of Triples
Three hub lncRNAs	14 (87.5%)	114 (87.69%)	238 (53.85%)
*RP5-1120P11*	9 (56.25%)	64 (49.23%)	92 (20.81%)
*DLEU2*	7 (43.75%)	77 (59.23%)	96 (21.72%)
*DDX11-AS1*	6 (37.5%)	45 (34.62%)	51 (11.54%)
GC-related ceRNA	16 (100%)	130 (100%)	442 (100%)

## Supplementary Materials

The following are available online at http://www.mdpi.com/2073-4425/9/6/303/s1, Figure S1: The strategy of integrative analysis of dysregulated lncRNA-associated ceRNA network in GC, Figure S2: PCC distribution in GC. (A) PCC higher the 95 percentile of the overall correlation distribution of lncRNA-mRNA pairs in GC. (B) PCC lower the 95 percentile of the overall correlation distribution of mRNA–miRNA pairs in GC. (C) PCC higher the 95 percentile of the overall correlation distribution of lncRNA–miRNA pairs in GC, Table S1: Differentially expressed lncRNAs, mRNAs, and miRNAs, Table S2: Potential ceRNA triples in GC-related ceRNA networks, Table S3: Functional enrichment analysis of ceRNA network. Table S4: The BC of all nodes in the ceRNA network.
