# Supplementary figures and images for: Integrative Analysis of Dysregulated lncRNA-Associated ceRNA Network Reveals Functional lncRNAs in Gastric Cancer
Source: Genes (Basel). 2018 Jun 18;9(6):303. doi: 10.3390/genes9060303 (PMC6027299; doi:10.3390/genes9060303)

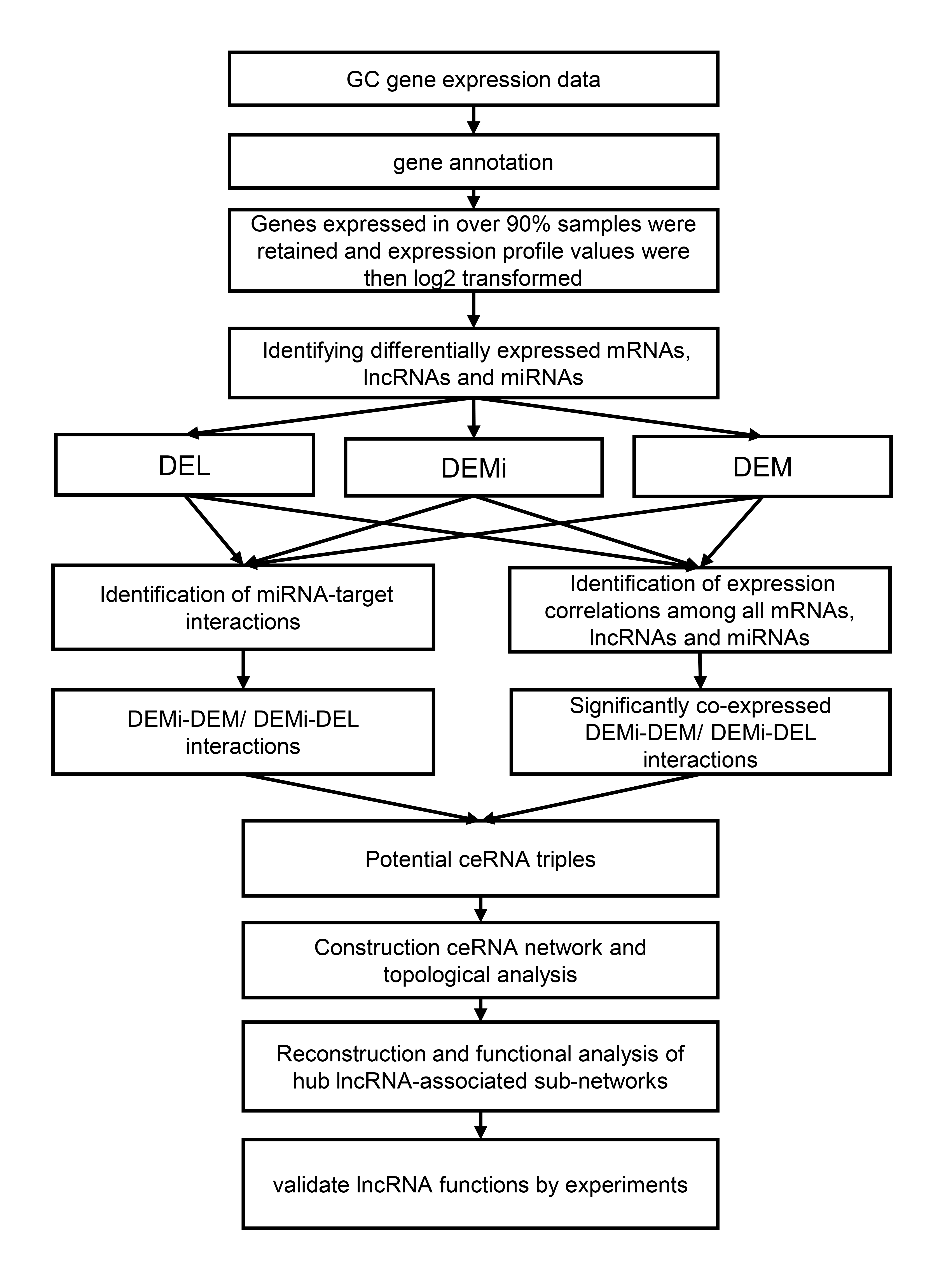

Supplement: Supplementary file 1 [file genes-09-00303-s001.zip › Figure S2 PCC distribution in GC.tif]

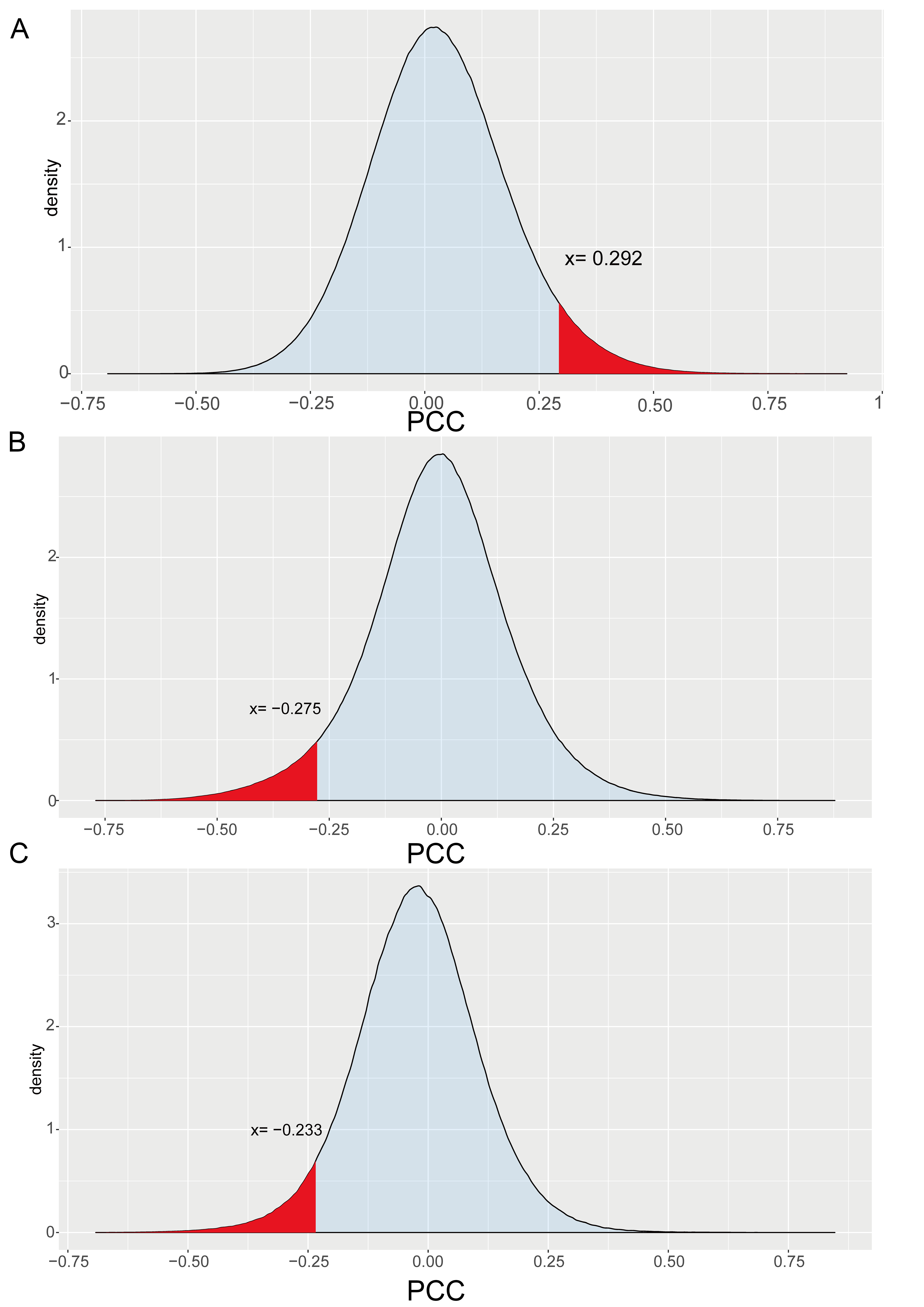

Supplement: Supplementary file 1 [file genes-09-00303-s001.zip › Figure S1 The strategy of integrative analysis of dysregulated lncRNA-associated ceRNA network in GC.tif]
